# The Expression of IL-6, TNF-*α*, and MCP-1 in Respiratory Viral Infection in Acute Exacerbations of Chronic Obstructive Pulmonary Disease

**DOI:** 10.1155/2017/8539294

**Published:** 2017-03-02

**Authors:** Jingtong Zheng, Yue Shi, Lingxin Xiong, Weijie Zhang, Ying Li, Peter G. Gibson, Jodie L. Simpson, Chao Zhang, Junying Lu, Jingying Sai, Guoqiang Wang, Fang Wang

**Affiliations:** ^1^Departments of Pathogenic Biology, College of Basic Medical Sciences, Jilin university, Changchun, China; ^2^School of Pharmaceutical Sciences, Jilin University, Jilin, China; ^3^Department of Respiratory Disease, Jilin Provincial People's Hospital, Changchun, China; ^4^Department of Disease Control and Prevention, Beijing Shunyi District Center, Beijing, China; ^5^Department of Respiratory and Sleep Medicine, John Hunter Hospital, Newcastle, NSW, Australia; ^6^Department of Respiratory and Sleep Medicine, University of Newcastle, New Lambton, NSW, Australia; ^7^Department of Intensive Care Unit, First Hospital of Jilin University, Changchun 130021, China; ^8^Department of Clinical Laboratory, The Second Hospital of Jilin University, Changchun 130021, China

## Abstract

Viral infection is a common trigger for acute exacerbations of chronic obstructive pulmonary disease (AECOPD). The aim of this study is to investigate the expression of cytokines in AECOPD. Patients with AECOPD requiring hospitalization were recruited. Meanwhile healthy volunteers of similar age that accepted routine check-ups and showed no clinical symptoms of inflammatory diseases were also recruited. Induced sputum and serum were collected. Induced sputum of participants was processed and tested for thirteen viruses and bacteria. Forty cytokines were assayed in serum using the Quantibody Human Inflammation Array 3 (Ray Biotech, Inc.). The most common virus detected in virus positive AECOPD (VP) was influenza A (16%). No virus was found in controls. Circulating levels of IL-6, TNF-*α*, and MCP-1 were elevated in VP and coinfection subjects (*p* < 0.05), while the levels of 37 other cytokines showed no difference, compared with virus negative groups and controls (*p* > 0.05). Additionally, VP patients were less likely to have received influenza vaccination. VP patients had a systemic inflammation response involving IL-6, TNF-*α*, and MCP-1 which may be due to virus-induced activation of macrophages. There are important opportunities for further investigating AECOPD mechanisms and for the development of better strategies in the management and prevention of virus-related AECOPD.

## 1. Background

Acute exacerbation of chronic obstructive pulmonary disease (AECOPD) is characterized by changes in sputum production, dyspnoea, and cough [1, 2]. AECOPD leads to significant morbidity and is among the major causes of death worldwide [2] and results in heavy societal and economic burden.

Viral infection has been confirmed as a cause of AECOPD and considered a major cause of AECOPD hospitalizations [3]. Viral detection may be influenced by the viral detection methodology and the sample that is used for assay. In several previous studies conducted with conventional methods (including viral culture and serology), the incidence of viral identification was underestimated when compared to molecular diagnostic methods [4–7]. With the development of molecular diagnostics, the detection of respiratory viruses in AECOPD is both easier and more accurate. Induced sputum that is obtained from the lower respiratory tract has relatively higher virus-detection rate when compared to samples from the upper respiratory tract [3].

The host inflammatory response is an important component of AECOPD. The profile of inflammatory markers may be used to classify airway inflammation in AECOPD with heterogeneous inflammation [8]. Many cytokines are involved in the development of COPD and the systemic levels of tumor necrosis factor-*α* (TNF-*α*) and interleukin-6 (IL-6) are higher in COPD than those of healthy controls (HC) [9–11]. However, expression of systemic inflammatory markers has not been well-studied in viral AECOPD. Assessing the relationship between AECOPD and the imbalance of inflammatory or anti-inflammatory mediators may assist in understanding the pathogenesis of AECOPD.

In this study, we aimed to determine the prevalence of several respiratory viruses in AECOPD and to detect the distribution of systemic inflammatory markers.

## 2. Methods

### 2.1. Patient Recruitment

Patients diagnosed with COPD and clinical symptoms of AECOPD requiring hospitalization were recruited from June 2012 to May 2013 in Jilin Provincial People's Hospital. Patients were recruited within 4–12 h after presentation. All of the patients were diagnosed with COPD according to the Global Initiative for Chronic Obstructive Lung Disease (GOLD) criteria (forced expiratory volume in 1 second (FEV_1_)/forced vital capacity (FVC) < 70%, and postbronchodilator FEV_1_ < 80%) [12]. AECOPD was defined with at least two major symptoms (increased dyspnoea, enhanced cough, or increased sputum production) or one major and one minor symptom (nasal discharge/congestion, wheeze, sore throat, and cough) for at least 2 consecutive days [13, 14]. Patients with a history of myocardial infarction, unstable angina, congestive heart failure, renal failure, cancer, pulmonary interstitial fibrosis, asthma, antivirus treatment, or currently active tuberculosis were excluded. In addition, COPD patients who were unconscious or declined to participate were also excluded. Meanwhile 12 healthy, never-smoking, nonatopic, similar-aged volunteers with no history of respiratory disease underwent sputum induction and lung function assessment. All of them accepted routine check-ups and showed no clinical symptoms of inflammatory diseases. Induced sputum and serum were collected and the following basic clinical characteristics were recorded and assessed: age, gender, smoking history, and lung function (GOLD levels). Written informed consent was obtained from the patients prior to the recruitment and this study was approved by the Human Research Ethics Committee of Jilin Provincial People's Hospital (approval number: 201405).

### 2.2. Exclusion of Samples

Sputum was taken to a sterile container and was analyzed immediately microscopically by Gram staining. After Gram staining and direct microscopic examination, sputum was incubated for 18–36 hours at 37°C in 10% CO_2_ by inoculating in eosin methylene blue agar, blood agar,* Brucella* agar, and chocolate agar media. Participants whose samples were identified as having bacterial infection or bacterial-viral coinfection were excluded.

### 2.3. Induced Sputum Samples

Recruited patients received nebulized 0.9% saline for 30 seconds [15]. Spirometry was repeated to measure the level of FEV_1_ every 5 minutes. Nebulization continued if the level of FEV_1_ had not fallen by more than 15% for a maximum of 20 minutes. All sputum was processed within 2 h. Mucus clumps were separated from saliva using a forceps, and a minimum of 200 *μ*L sample was transferred to a test tube; 4 times the volume of Sputolysin (Sigma, Poole, UK) working solution was added to the tube and was mixed at room temperature for 30 minutes. Subsequently an equal volume of PBS was added and fully mixed. The samples were then filtered and centrifuged at 400 ×g for 10 minutes. After removing the supernatant, the cell pellet was resuspended in Lysis Buffer (Qiagen, Crawley, UK). Later total nucleic acid extraction was performed in 200 *μ*L sample suspended in Lysis Buffer.

### 2.4. Serum Samples

10 mL venous blood was collected into a heparin-lithium anticoagulant tube and the serum was stored at −80°C for later analysis of inflammatory markers. All samples were obtained within 24 h of enrolment.

### 2.5. Detection of Respiratory Viruses and Bacteria

Measurement of virus was performed on RNA by Viral RNA Mini Kit (QIAamp®). RNA was extracted from induced sputum from AECOPD participants and controls. Thirteen viruses were tested, using RT-PCR (ABI AgPath-ID™ One-Step RT-PCR Kit) to detect influenza A and B; RT-PCR (Super Script® III One-Step RT-PCR System with Platinum® Taq DNA Polymerase, Invitrogen) to detect hMPV, coronavirus, adenovirus, and bocavirus; Multiplex RT-PCR (Super Script III One-Step RT-PCR System with Platinum Taq DNA Polymerase and Platinum PCR Super Mix, Invitrogen) to detect parainfluenza −1, −2, −3, and −4, rhinovirus, respiratory syncytial virus, and enterovirus (Ev71). Sputum was used for bacterial culture as well. According to the results, participants were divided into virus positive AECOPD (VP) participants, bacteria positive AECOPD (BP) participants, virus-bacteria negative AECOPD (VN) participants, coinfection (CI) participants, and controls.

### 2.6. Profiling of Inflammatory Markers

Serum was obtained after centrifugation (3,500 rpm for 20 min) of whole blood and was kept at −80°C prior to inflammatory marker profiling. The levels of cytokines were evaluated by Quantibody Human Inflammation Array 3 (Ray Biotech, Inc.), according to the manufacturer's instructions.

### 2.7. Statistical Analysis

Normality of data was tested by the Kolmogorov-Smirnov test. For basic characteristics of participants in Table 3, difference between the two groups was analyzed by Chi-square test or student's *t*-test. For cytokine expression assay, data were of normal distribution, ANOVA was used to evaluate the expression of different cytokines, and the levels were measured through the multiple comparison method. Data were expressed as mean values ± standard error of mean. Statistical analysis was performed using Graph pad 6.0 and *p* < 0.05 was considered statistically significant.

## 3. Results

### 3.1. Patient Characteristics

**Figure 1:**
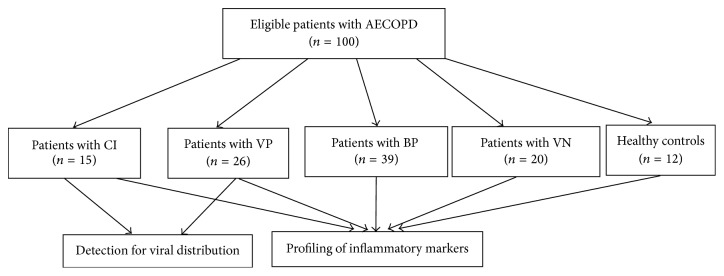
Strategies for screening patients with viral AECOPD.

There were 100 eligible AECOPD patients during June 2012 to May 2013. The recruitment process is shown in Figure 1. There were 26 virus positive (VP) patients, 39 bacteria positive (BP) patients, 15 coinfection (CI) patients, and 20 virus negative (VN) patients. The severity levels of 4 patients (4%) were classified as GOLD I, 39 (39%), GOLD II, 52 (52%), GOLD III, and 5 (5%) GOLD IV (Table 1). 41 (41%) participants were vaccinated against influenza in the previous year. Almost all (97%) participants had a history of smoking and 38 (38%) were current smokers. In the current study twelve healthy volunteers were also recruited.

### 3.2. Detection of Respiratory Viruses and Bacteria

**Figure 2:**
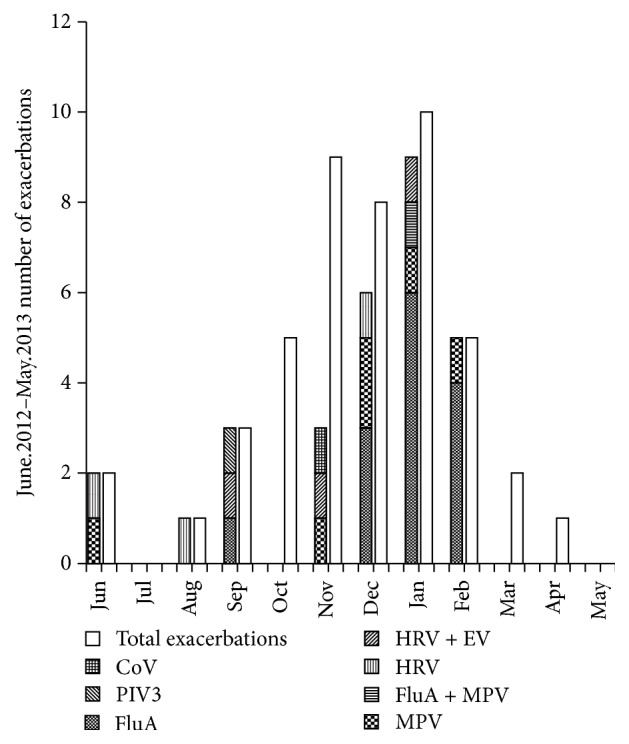
Monthly distribution of viruses detected by multiplex PCR and RT-PCR in AECOPD. FluA: influenza A, MPV: human metapneumovirus, HRV: human rhinovirus, EV: enterovirus, PIV3: parainfluenza type 3 virus, CoV: coronavirus.

The most common virus was influenza A (10 cases, 10%), and the most common bacterium is* Haemophilus influenzae* (Table 2). The number of AECOPD in winter and spring was larger than that in summer and autumn (Figure 2) (*p* < 0.05). No AECOPD were observed in May and July, and no VP were observed in March, April, May, and July.

### 3.3. Correlations between Viral Infection and Clinical Symptoms

Baseline characteristics and treatment of the recruited patients are shown in Table 3 according to whether they are VP, VN, or CI. Analysis suggested no differences between VP and VN in terms of runny or congested nose and discolored sputum (*p* > 0.05) (Table 3). VP participants more frequently had a fever (38% versus 15%, *p* < 0.05) and sore throat (46% versus 20%, *p* < 0.05) when compared with VN participants. Moreover, CI participants had the highest rate of fever (40%) and sore throat (46%).

The rate of current smokers in CI participants (40%) and VP participants (35%) was higher than VN participants (30%) (Table 3). 11 (42%) VP participants were reported prior hospitalization for AECOPD, which had a higher rate than CI participants (40%) and VN participants (30%). The rate of influenza vaccination for that season was less in VP participants (23%), compared to VN participants (80%; *p* < 0.05). The number of participants with exacerbation symptoms that lasted for more than 4 days was 70% in VP, which was higher than that of VN (*p* < 0.05). The percentages of VP participants that received treatments showed no difference when compared with that of VN participants (*p* > 0.05).

### 3.4. Profiling of Inflammatory Markers

**Figure 3:**
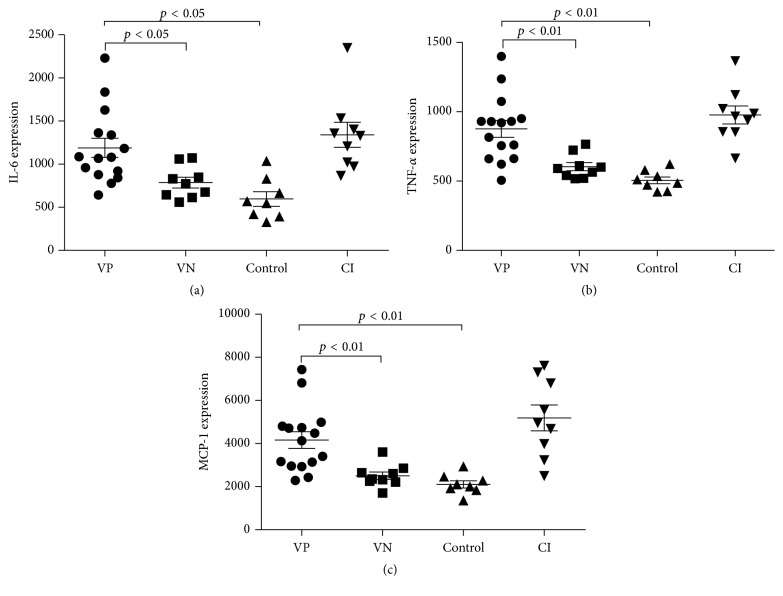
(a) The overexpression of IL-6 between different groups. (b). The overexpression of TNF-*α* between different groups. (c). The overexpression of MCP-1 between different groups.

Forty measured markers are shown in Table 4. The results of Quantibody Human Inflammation Array showed that the levels of IL-6, TNF-*α*, and MCP-1 in VP and CI were higher than those in HC (*p* < 0.01), and overexpression of IL-6, TNF-*α*, and MCP-1 was also observed between VP and VN (*p* < 0.05) (Figure 3). The differences in the levels of IL-6, TNF-*α*, and MCP-1 between VN and HC were not significant (*p* > 0.05), and the levels of these three markers demonstrated no difference between VP and CI (*p* > 0.05).

## 4. Discussion

Studies specifically related to viral infection and cytokines expression in AECOPD are uncommon, especially in Asia. To the best of our knowledge, this is the first study conducted in the Alpine region in Asia, in relation to the prevalence of respiratory viruses and the expression of cytokines for AECOPD.

Interestingly, the viral distribution in our study is also quite different from most of other studies. Influenza A was the most common respiratory virus in our study and was found in 10% of AECOPD subjects (Table 2), which is consistent with a finding in which the most common virus identified was influenza A (31%) in 2069 tested virus positive nasopharyngeal aspirate samples between August 2014 and July 2015 in Australia [16]. This contrasts with other reports. The detection rate of influenza A in Hong Kong was 7.3% [17]. Prior influenza vaccination can modify the influenza infection rate.

In our study, 29% of recruited AECOPD patients were vaccinated against influenza last year, and among these, 80% had been vaccinated in VN, 46% had been vaccinated in CI, and however only 23% participants were vaccinated in VP. Cohorts with higher vaccine coverage for influenza have been found to have a lower prevalence of influenza [18, 19], which is supported by our comparison of VN versus VP. Moreover, population with low influenza vaccine coverage had higher influenza detection rates, and none of the influenza-positive patients received vaccination in the previous year [20]. Considering the extremely low influenza vaccination coverage (1.9% of the total population) in China and the factor that influenza-positive rate is higher in adults and elderly [21], attention should be paid to the rapidly aging country to effectively control influenza A infection. Further investigation is needed to confirm the exact reasons for the variance in viral distribution and viral detection rates between our findings and other studies.

We also note the odd phenomenon that RSV was not found in viral AECOPD in our study all the year round. In a study conducted from January to June 2014 in China, despite low rate, three cases of RSV infections were found in nasopharyngeal samples of 81 participants admitted for AECOPD [22], which was inconsistent with our finding. Furthermore, RSV is an important cause of viral infections in lower respiratory tract in elderly and high-risk adults [23] and is ranked as one of the most prevalent viruses in a total of 1728 AECOPD subjects in nineteen studies in a systematic review [24]. The reason why RSV was not observed in our investigation remains elusive and needs further studies.

Viral symptoms include increased rhinorrhoea, sore throat, and fever/chills [25]. Besides, sore throat has greater specificity for viral infection than rhinorrhoea. Fever and sore throat were the two major symptoms which differentiated viral AECOPD from nonviral AECOPD (Table 3), which suggests that the diagnosis of viral symptoms may be the elementary step to accurate case-definition of viral AECOPD. The combination of clinical symptoms with measurement of inflammatory marker levels markedly increased the predictive accuracy compared with the diagnosis of clinical features alone [26].

The profiling of 40 inflammatory markers was conducted to assess the association between viral infection and host cytokine responses in AECOPD. In previous studies the major objective of detection was to investigate the types of microorganisms involved in AECOPD, and less emphasis was placed on proinflammatory cytokines. We found that patients with viral infections showed higher levels of TNF-*α*, IL-6, and MCP-1 than those without (Figure 3). Patients with a coinfection by virus + bacteria also exhibited higher levels of TNF-*α*, IL-6, and MCP-1 than those with negative microbiology results. Furthermore, the expression of these markers showed no difference between VN and HC (*p* > 0.05), indicating that the difference of marker expression might be caused by viral infection. IL-6 is a pleiotropic acute-phase cytokine involved in the host immune response to infection, reflecting the severity of lower airway inflammation [27, 28]. Higher levels of IL-6 are associated with the exacerbations of COPD and increase the predictive accuracy of viral infection when combined with the clinical diagnosis [26, 29]. TNF-*α* is a mononuclear-phagocyte-origin cytokine that has pleiotropic effects on innate host responses to microbes [30] and it recruits inflammatory cells into the site of infection [31] and is overexpressed in AECOPD [32]. MCP-1 is a potent chemoattractant that contributes to the recruitment of inflammatory cells into the site of infection [33, 34]. Higher levels of MCP-1 have been reported in patients with virus-induced respiratory illness [35, 36]. There is increasing evidence that macrophages orchestrate the inflammatory responses of COPD through the release of chemoattractant and proteases [37]. Higher levels of IL-6, MCP-1, and TNF-*α* may be associated with the activation of macrophages. Influenza virus, which was the most common virus in our study, induces the activation of macrophages via Toll-like receptor 3 (TLR3) [38]. Yageta et al. [39] found that isolated murine macrophages were activated by costimulation of cigarette smoke extract and influenza A virus. In response to influenza A virus-infection, macrophages produce large amount of cytokines such as IL-6, TNF-*α*, and MCP-1 [40–43]. Tissue macrophages are the key cell types responsible for the production of antiviral and immunoreactive cytokines [44]. Taken together, we conclude that higher levels of those inflammatory markers might be induced by viral inflammation and activation of macrophages, which might provide support for further investigation in the pathogenesis of viral AECOPD in Asia.

In summary, we have established that viral infection is common and has an altered viral profile in AECOPD that occurs in Alpine China. Viral AECOPD is associated with a systemic immune response that involves IL-6, TNF-*α*, and MCP-1, suggesting a prominent role for macrophage activation in AECOPD. Future work can define the clinical and treatment implications of these result, and lay the basis for further investigating the role of immune system in the pathogenesis and development of AECOPD.

## 5. Conclusion

Influenza A is the most prevalent viral pathogen in AECOPD in Northern China. Patients with viral AECOPD were less likely to have received influenza vaccination. They had a prominent systemic immune response involving IL-6, TNF-*α*, and MCP-1 which may be due to the activation of macrophages that was caused by the viral infection. There are important opportunities for further investigation of AECOPD mechanisms and for the development of better strategies in the management and prevention of AECOPD that is associated with viral infection.

## Figures and Tables

**Table 1:** Clinical characteristics of AECOPD.

Category/parameter	Total (*n* = 100)
*Sociodemographic data *	
Age mean value ± SD, years	70.41 ± 11.75
Male *n* (%)	62 (62%)
Current smokers *n* (%)	38 (38%)
Current or former smokers *n* (%)	97 (97%)
Influenza vaccination *n* (%)	41 (41%)
Acute COPD exacerbation in last year *n* (%)	75 (75%)
Duration of COPD	12.95 ± 0.93
Hospitalization in last year *n* (%)	11 (11%)
*COPD severity as per GOLD n (%)*	
GOLD I (mild)	4 (4%)
GOLD II (moderate)	39 (39%)
GOLD III (severe)	52 (52%)
GOLD IV (very severe)	5 (5%)

**Table 2:** Type distribution of detected respiratory viruses and bacteria.

Types	Percentage (*n* = 100, 100%)
Influenza type A	10 (10%)
hMPV	6 (6%)
Rhinovirus	4 (4%)
Enterovirus	3 (3%)
Human parainfluenza type 3	2 (1%)
Coronavirus	1 (1%)
Influenza A + hMPV	2 (2%)
Rhinovirus + enterovirus	3 (3%)
*Haemophilus influenzae*	15 (15%)
*Pseudomonas *spp.	8 (8%)
*Streptococcus pneumoniae*	5 (5%)
*Moraxella catarrhalis*	4 (4%)
*Staphylococcus aureus*	4 (4%)
Other bacteria	3 (3%)
Rhinovirus + *S. pneumoniae*	6 (6%)
Rhinovirus + *H. influenzae*	5 (5%)
Rhinovirus + *Pseudomonas *spp.	3 (3%)
Adenovirus + *S. pneumoniae*	1 (1%)

**Table 3:** Baseline characteristics according to viral status.

	VN *n* = 20	VP *n* = 26	CI *n* = 15
Male, *n* (%)	12 (60%)	16 (62%)	9 (60%)
Current smoker	6 (30%)	9 (35%)	6 (40%)
Total cigarette consumption (pack years)	59^*∗*^	61	58
Prior COPD hospitalization	6 (30%)^*∗*^	11 (42%)	6 (40%)
Influenza vaccinations	16 (80%)^*∗*^	6 (23%)	7 (46%)
Exacerbation symptom present ≥ 4 days	8 (40%)^*∗*^	18 (70%)	5 (33%)
*Symptoms at entry*			
Fever	3 (15%)^*∗*^	10 (38%)	6 (40%)
Sore throat	4 (20%)^*∗*^	12 (46%)	7 (46%)
Runny or congested nose	9 (45%)	17 (65%)	9 (60%)
Discolored sputum	8 (40%)	16 (62%)	8 (53%)
*Treatment*			
Oxygen therapy	2 (10%)	3 (12%)	3 (20%)
Antibiotic therapy	11 (55%)	15 (58%)	8 (53%)
Corticosteroids	5 (25%)	6 (23%)	6 (40%)

The difference between the two groups was analyzed by Chi-square test.

^*∗*^ *P* < 0.05 versus the virus positive AECOPD.

**Table 4:** Forty inflammatory markers measured by Quantibody Human Inflammation Array.

POS1	POS2	BLC
Eotaxin	Eotaxin-2	G-CSF
GM-CSF	I-309	ICAM-1
IFN*γ*	IL-1*α*	IL-1*β*
IL-1ra	IL-2	IL-4
IL-5	IL-6	IL-6sR
IL-7	IL-8	IL-10
IL-11	IL-12p40	IL-12p70
IL-13	IL-15	IL-16
IL-17	MCP-1	MCSF
MIG	MIP-1*α*	MIP-1*β*
MIP-1*δ*	PDGF-BB	RANTES
TIMP-1	TIMP-2	TNF-*α*
TNF *β*	TNF RI	TNF RII

## Abbreviations

COPD: Chronic obstructive pulmonary disease
AECOPD: Acute exacerbations of chronic obstructive pulmonary disease
GOLD: The Global Initiative for Chronic Obstructive Lung Disease
hMPV: Human metapneumovirus
Ev71: Enterovirus
IL-6: Interleukin-6
MCP-1: Monocyte chemoattractant protein-1
TNF-*α*: Tumor necrosis factor-*α*
VP: Virus positive AECOPD
VN: Virus-bacteria negative AECOPD
HC: Healthy control.
